# Novel instrumentation in urologic surgery: Shock wave lithotripsy

**DOI:** 10.4103/0970-1591.70780

**Published:** 2010

**Authors:** Michelle J. Semins, Brian R. Matlaga

**Affiliations:** James Buchanan Brady Urological Institute, The Johns Hopkins University School of Medicine, Baltimore, MD, USA

**Keywords:** Novel instrumentation, shock wave lithotripsy, urolithiasis

## Abstract

Extracorporeal shock wave lithotripsy (SWL) was first introduced in 1980 and it rapidly revolutionized the treatment of stone disease. SWL is a non-invasive, outpatient procedure that now accounts for the majority of stone removal procedures. Since the introduction of first generation lithotripter, the Dornier HM3 machine, SWL devices have undergone many modifications secondary to limitations, in efforts to create a more effective and efficient way to treat stones and decrease possible morbidities. Herein, we review the evolution of the technology and advances in the instrumentation over the last three decades.

## INTRODUCTION

Extracorporeal shock wave lithotripsy (SWL) was first introduced in 1980 and it rapidly revolutionized the treatment of stone disease. Prior to the SWL era, patients harboring upper urinary tract calculi often required invasive and morbid surgical procedures to effect stone removal. Following the introduction of SWL, renal and ureteral stones could be treated in a non-invasive, outpatient fashion. As a result of this paradigm shift in stone treatment, SWL became widely adopted. Recently, SWL reported to account for as many as 69% of all stone removal procedures.[1]

The first generation lithotripter to be widely distributed was the Dornier HM3 device. Although initially there was great excitement that accompanied the introduction of this device, as it represented a novel and non-invasive method of treating patients with upper urinary tract calculi, soon thereafter it was realized that there were also limitations to this technology. Large stone burdens, certain stone compositions, and specific stone locations were all factors that were found to affect the success of SWL. With increasing experience with SWL, it was found that shock waves may cause renal parenchymal damage, with potential long-term consequences. Finally, the Dornier HM3 was considered a cumbersome and immobile device.

In the three decades since its introduction, SWL has gone through a number of evolutions, with later generation devices aiming to make SWL a more convenient and effective intervention. Herein, we will review advances in the instrumentation and technology of SWL over the last three decades.

## EVOLUTION OF THE INSTRUMENTATION AND TECHNOLOGY

The initial lithotripter approved by the United States of America Food and Drug Administration was the Dornier HM3. As later generations of lithotripters were developed and introduced in the market, changes were made to device attributes such as the shock wave source, coupling mechanisms, machine size, imaging and targeting capabilities, and focal zone parameters. These changes were made with the goals of improving stone fragmentation and reducing tissue injury, while also simplifying the convenience and efficacy of the technology.

### Shock wave sources

The original Dornier device was termed an electrohydraulic lithotripter, meaning that the shock wave was generated by a spark gap electrode which was seated in the lithotripter water bath. To generate the shock wave, a capacitor is charged; as it discharges, it causes an explosion at the electrode (spark plug) which vaporizes the water between the electrode tips. This burst of plasma, or energy, produces a spherical shock wave, which is focused when it encounters the ellipsoid reflector. As the shock waves are generated over the course of a treatment session, the electrode tips progressively erode.[2] As these tips erode, the shock wave can become more variable and less predictable.[3] From a practical standpoint, the electrode can only produce several thousand shocks before it needs to be replaced. Electrode technology has advanced, though, such that present day electrodes are encapsulated or self-advancing, which results in greater shock wave consistency and electrode lifespan.

In part, as a response to the limited lifespan and inherent variability of the spark gap energy source of electrohydraulic lithotripters, a number of manufacturers investigated alternative shock wave sources. Electromagnetic shock wave generators were designed to overcome many of the shortcomings associated with electrohydraulic lithotripters. Although there are several different designs of electromagnetic lithotripters, all of the electromagnetic shock wave generators consist of a coil that is either on a flat surface with a conductive membrane on top, wrapped around a cylinder, or on the inner surface of a spherical cap. As with electrohydraulic technology, a capacitor initiates the discharge but in this case it produces a magnetic field which repels a membrane and results in a shock wave that is then focused with either an acoustic lens, a parabolic reflector or is focused at initiation. Electromagnetic sources are more consistent, reproducible and durable than electrohydraulic generators, with reported lifespans of one to two million shock waves.[2]

Although not in widespread use at the present time, piezoelectric shock wave sources have also been used in SWL. However, clinical results with piezoelectric devices are reported to be inferior to those achieved with electrohydraulic and electromagnetic devices. Piezoelectric generators produce a shock wave when a capacitor is discharged through an array of piezoceramic elements positioned on a reflector.[4] As with electrohydraulic and electromagnetic shock wave generators, piezoelectric systems generate a shock wave by the phenomenon of non-linear propagation.[2]

### Size of the machines

The Dornier HM3 lithotripter was a sizable apparatus, consisting of a large water bath and patient gantry, and occupied a large footprint of hospital floor-space. As a result, the HM3 was non-transportable, and its utilization was limited to only those physicians with a proximity to its location. As later generations of lithotripters were developed, one overarching goal was to reduce the size of the device and make it more transportable. In modern practice, lithotripters have become much smaller and transportable, allowing a wider population of patients and physicians to have access to this treatment approach.

### Coupling mechanisms and treatment heads

The Dornier HM3 lithotripter is a water bath lithotripter, meaning that the patient is immersed in a bath of degassed water during the treatment session. The underwater environment of the HM3 provides an excellent acoustic coupling medium, as shock waves travel from the electrode through the water environment and into the patient with a minimal loss of energy. Although there are a small number of lithotripters presently in use, which rely on a complete or partial water bath for coupling the patient to the shock wave source, the majority of modern devices use dry treatment heads. In these cases, the patient is coupled to the lithotripter with either gel or oil solutions, which are placed on the treatment head and the patient is placed to the device acoustically. The transition from water bath designs to dry head treatment devices can be viewed as advancement in technology, as it allowed for the development of smaller and transportable machines. With dry treatment heads, however, air pockets can form in the coupling medium as the patient is joined to the lithotripter. These air bubbles can have an adverse effect on the success of SWL, as they impair the transmission of shock wave energy from the lithotripter to the patient. The effect of even a small amount of air bubbles within the coupling medium can have a dramatic effect on treatment efficacy: air pockets of just 2% of the coupling interface reducing the stone breakage by 20–40%.[5,6] In the water bath design, such concerns did not exist, as there were no coupling defects when the patient was submerged along with the shock wave source.

Recently, lithotripters with two treatment heads have been developed. These dual head machines have two shock sources that fire along different paths. Depending on the programming design, the treatment heads can fire in a simultaneous or alternating fashion. Theoretical advantages with dual head lithotripters include the potential to reduce treatment time, as well as the ability to enhance stone fragmentation.[7–10] Studies of dual head lithotripsy in a porcine model have demonstrated that renal damage is no greater than that encountered during conventional SWL.[11,12] However, in the case of electrohydraulic dual head lithotripters, electrode wear can begin to affect the timing of shock wave delivery which can affect treatment outcome.

### Imaging and targeting capabilities

The HM3 lithotripter relies on a system of biplanar fluoroscopy for localizing and targeting the stone. With second generation machines, both ultrasound and fluoroscopy can be integrated into the lithotripsy system such that combined targeting can be performed using both the imaging techniques. Ultrasound may provide a number of benefits over fluoroscopy: no exposure of patient or provider to ionizing radiation, ability to target radiolucent stones, and real-time monitoring. However, ultrasound is inferior to fluoroscopy in the identification of ureteral stones, targeting stones in obese patients, and is also a more technically difficult modality to learn to operate.

### Focal zone width and pressures

The focal zone of the lithotripter is the region of high acoustic pressure which is located about the device’s focal point – the point in space at which the shock wave pulse is focused. During a lithotripsy treatment session, the target stone is placed at the focal point, and efforts are made to ensure that the stone stays at the focal point or at least within the focal zone. As lithotripter technology advanced, attention was turned to modifications of the lithotripter focal zone, a process that attempted to optimize stone fragmentation while minimizing tissue energy. A wide focal zone is desirable in some respects, as it will maximize the likelihood of the stone receiving an effective dose of shock wave energy with each lithotripter pulse. However, a wide focal zone also exposes a larger amount of renal parenchyma to high acoustic energy, which may increase the deleterious tissue effects of shock wave energy. Additionally, a wide focal zone may increase the pain associated with SWL. Discomfort associated with SWL is primarily due to the sensation of cutaneous pain over the area of entry shock wave energy. With a closed lithotripter aperture, the area of cutaneous shock wave entry is narrow, which results in increased pain at the entry site as well as a wide focal zone. Opening the lithotripter aperture has the opposite effect.

As a result, later generations of lithotripters were developed with narrow focal zones; for example, the Dornier HM3 had a focal zone of 15 by 60 mm, whereas some modern devices have focal zones only 5 mm in maximal diameter.[3] As the focal zone narrows, peak pressure generated within the focal zone rises, in some cases to pressures far above those achieved with the Dornier HM3. In many cases, though, altering the acoustic profile of the lithotripter to narrow the focal zone did not have the desired enhancement of stone fragmentation. One reason may be that stones are a moving target during a lithotripsy session; their position in the body varies with respiration. With a small focal zone, accurate targeting can be more difficult, and the stone may not receive an adequate number of shock waves to effect fragmentation. Additionally, even though later devices were designed to be used without anesthesia, SWL remains a procedure that requires, at minimum, some degree of sedation in order to maintain a non-mobile patient. In an effort to balance the relative advantages and disadvantages of variously sized focal zones, the marketplace is now seeing the introduction of lithotripters in which the physician can control focal zone size. Research has shown that the optimal settings for the most efficient fragmentation and minimal renal damage are likely a wider focal zone with lower pressures.[13–15]

## CURRENT AND FUTURE RESEARCH

Although the past three decades have witnessed a number of great advances in our understanding of shock wave technology and its application to stone fragmentation, there remain a number of areas rich for continued development. Dual pulse techniques, whether from two separate shock wave treatment heads or from a single treatment head in a tandem fashion, have been recently described.[8,16–18] Although preliminary data relying on *ex vivo* and animal models are encouraging, further work can be done to better understand the clinical benefits of such technology. Our improved understanding of the acoustic coupling process during SWL has helped to maximize the efficiency of devices presently in use. However, future lithotripter designs may be able to provide real-time feedback on the coupling process, or even utilize a novel coupling technique.

Although both ultrasound and fluoroscopy permit the effective targeting of a stone for SWL, imaging modalities can still be advanced. At present, the shock waves are generated whether or not the stone is in the lithotripter’s focal zone. A continuous feedback mechanism may be able to control the administration of shock waves by limiting the firing of the lithotripter to only when the stone is residing in the focal zone. Additionally, in many cases, the presently available imaging modalities provide only limited information to the physician with regard to treatment endpoint. As a result, some patients undergoing SWL are likely to be overtreated with shock waves, as it is difficult for the physician to know when a stone has completely fragmented. Future imaging modalities may better assess the completeness of stone fragmentation, making the decision as to when to terminate a lithotripsy session less confusing.[3]

## CONCLUSION

SWL is a highly useful technology which has revolutionized treatment of stone disease. The exact mechanism of stone fragmentation and long term effects of renal injury are not fully understood. SWL technology has helped many stone patients but there remains a group which do not benefit with this treatment. Future modifications in shock wave generations, adjustment of energy levels, better focussing techniques and coupling may improve the outcome of lithotripsy.
